# Superior Court of Buffalo

**Published:** 1866

**Authors:** 


### Superior Court of Buffalo.

OLIVER J. OSTRAM, *against* PETER TERTIUS KEMPSON. March Civil Trial Term. Before Hon. Isaac A. Verplanck and a jury. March 12, 1866.

JOHN D. HILL having been sworn testified: Have been a practicing physician and surgeon for seventeen years; reside in Buffalo; have examined Mrs. Ostram's arm and wrist, and found it had been injured; my impression, from the examination is, that there was a fracture of the lower end of the radius, known as "Barton's Fracture," usually within an inch of the articulation; it is possible there might have been a dislocation of the ulna, or there might not have been; there is a projection there which might have occurred from inflammation; I think a surgeon could have told at time of injury whether it was fracture or dislocation; it was probably an oblique fracture; I think this could have been told by a surgeon at time of injury; it usually can; there is no evidence that there was a dislocation of the joint; dislocation of the wrist joint is not as common as fracture; fractures of the radius are common; dislocations of wrist joint without fracture are not common; I believe I have seen one out of a dozen or fifteen cases; no difficulty in ascertaining whether it is a fracture or a dislocation in the first stages; if my idea is correct the usual way of treating, or the way I treat is, to put the arm half way between supination and pronation; it keeps the bones in a line with each other; *it should not be laid flat because it brings bones cross-wise, and draws the radius down and throws the ulna out, producing greater deformity.* The position I have named keeps them in place, dropping the hand towards the ulna side, the extension muscles of the thumb, which are attached to the radius, prevent the muscles from drawing the radius back. *I have always used the crooked splint, either the Bond splint or the Day splint;* the inside splint is a wedge splint to keep the shafts separate. I should put a bandage around the hand extending to the wrist, then splint and bandage up to elbow; splint should be wide enough so that bandage would not press upon the arm, that is all. I direct patient to keep arm on pillow or in a sling, hand protruding through sling so that the weight of the hand would drop on the ulna; if there be great inflammtion let the arm rest on pillow and not in sling, and if painful loosen bandages. I should clip the edges of bandages, but would not take it off unless absolutely necessary, under a fortnight or longer; keep arm in that position, giving attention to indications. In patient of Mrs. Ostram's age arm should be kept in spints from forty to sixty days; in case of fracture consolidation of the bone will have taken place in that time sufficient to retain fracture in its position without aid of splints. Change dressing once a week; this I usually do; this may not always be necessary, but as a caution; the condition we expect when splints are removed is, that bones are in place and the arm in normal condition, with some slight enlargement and a temporary stiffness of the hand. I should expect motion all the time, but not to great extent for some weeks, might be six to twelve weeks in ordinary cases; if at end of that time there was no motion of fingers I should suppose there had been inflammation of the flexor tendons, producing adhesion. At twelve or fourteen weeks if I found this condition with large projection of ulna, I should suppose inflammation with a dislocation of ulna from its articulation with radius, and that it was still out of place. *It would not be good practice in case of fracture of radius to dress on a straight splint*, with hand flat, for the reasons I have given before; I would in simple dislocation adjust dislocation and dress on straight splint; the other way would not be necessary. In fractures we reduce parts to their places. [Mrs. Ostram is produced and witness examines wrist and says:] I think the radius approaches the ulna, making arm crooked; there is also a projection of the head of the ulna; it may have been produced by partial dislocation of its articulation with head of radius, or it may have been produced by inflammation of the articulations making a deposit between the articulating surfaces of the radius and ulna. The radius is thrown down on the ulna that carries the tendons that play through a sheath on the wrist to the finger, and thus draws the fingers out of line, instead of closing them; it draws them against the thumb instead of the palm of the hand; I think there has been inflammation of the sheath and tendons not sufficient to prevent flexion; the effect is loss of use of it, do not say entire loss; its usefulness is greatly impaired. If at end of eight weeks I found swelling and a want of use I should infer the case was imperfect and should try to get motion in fingers; should use embrocations, probably could do nothing by way of re-adjustment after the eighth week. The appearance of the arm shows displacement; don't know but it might occur with any one, but I never saw a case when treated with pistol splints. The cases of this kind that I have seen were treated with straight splints; I have known such results from straight splints, what I call improper treatment; I do not remember to have had a case where patient was as old as Mrs. Ostram; have had them at fifty years of age; I suppose after fifty there is greater liability, less recuperative power in nature; if cure is complete it takes a long time; no more difficulty in keeping bones in place in old than young. If she has had fingers become eventually flexible I should think it favorable to further recovery.

*Cross-examined.*—My business is principally confined to family practice. The splint I have spoken of is the pistol splint; can't tell how long it has been in use; it was used when I commenced practice; I do not think there is any difference in the profession about the use of straight or pistol splints; have heard surgeons speak of using straight splints, but know of no authority for it. HAMILTON is an authority among a portion of the profession; think probably he is; don't know whether he sanctions use of straight splints; never read the work on that subject—have read portions of it; I don't know any authority by name of "*Smith on Fractures;*" never read his work; have read some of Dr. Mott's writings; don't call to mind what he says about this particular fracture; don't know if he endorses straight splint; I don't call any recent author to mind who says anything in regard to straight splints. This was what is called the "Barton Fracture," which includes any fracture which comes within one or one and a half inches of articulation of carpus. I cannot tell the direction of the fracture; all I mean to say, it was an oblique fracture. This fracture did not necessarily imply a displacement of the ulna nor of the carpus; I call the end of the radial bones at either extremity the head. The styloid process may or may not have been broken; in injuries of this kind there is a bulging at once; it becomes large at once—large on the palmer side; it is caused by a displacement; this is caused by the action of the muscles; it is apparent on the dorsal side, but not in the same degree; it is not swelling from inflammation, which may not take place in twelve or twenty-four hours; the arm over the seat of fracture would perhaps be tender at once or in a few hours; if puffing is caused by fracture it will subside soon; if sprain, it may not subside. I have never seen a case where it was difficult to determine nature of injury. Having reduced the fracture I would apply a bandage round the fingers and to the wrist over the head of the radial bone—not beyond the radial bone; some carry bandage nearly to elbow: this I think immaterial; if I used Bond's splint I should do it; if the pistol, with wedge inside splint, I should not; this bandage being on I then apply splint and bandage arm permanently; the object of the pistol splint is to dress the hand toward the ulna side of wrist. If there was a fracture of the radius and a very considerable displacement of the ulna this would be proper; this would not drive the ulna into the carpal bones and produce inflammation; it would hold both ulna and radius in place. The natural condition of the hand is straight on a line; whether the part injured should be placed in a natural position depends on the nature of the injury and the tendency to displacementof the fractured bone by muscular contractions; bandaging alone will not control the muscular action of the hand and wrist; it will impede the flexor muscles of the hand and wrist, but not control them perfectly, but may imperfectly; after taking off the bandages there is always anchylosis present in a greater or less degree; the recovery is gradual, depending on the age of patient and other conditions; have frequently seen it when patient could not shut the hand when bandage was removed; cannot tell how long I have known it to continue; don't remember that books state cases where it has continued from *one* to *five* years; I think books state that recovery is from one to six months; don't remember what Hamilton, Smith or Mott say on that subject. I intend to keep posted; Hamilton and Smith are late works on this subject. Have read several articles in American Medical Science, and Prof. Gross' work; this I read more or less every week. Dr. Weiss had a case like this; treated with straight splints; can't tell age of patient; saw a case six or seven, may be more years since; patient middle age; hand not useful; can't call to mind another case of this kind; that case was similar; don't remember habits or age of patient; can't tell how many cases of fracture of radius I have had; I have now ten cases under treatment; one middle age, one past middle age; never have known an unfortunate result from use of pistol splint. In determining results we must consider age of patient, their manner of life, their own care in absence of surgeon. If patient makes efforts to use hand too early it is injurious; loosening bandage by patient is improper; these things would tend to counteract efforts of best surgeons. I do not think this fracture would tend to produce an inflammation of the point where the bones articulate with the carpus; there would be some tension of the muscles. If the fracture was occasioned by falling on back of hand there would be more tension; this would continue longer before recovery in a person of Mrs. Ostram's age than in a middle aged person; if the fall was on the dorsal side of the hand it would produce more of inflammation than on the other; it would be more likely to produce dislocation; it would increase liability to inflammation; it might occur and the inflammation be kept in reasonable bounds; there would be considerable inflammation. If there was a dislocation of the wrist its surrounding ligaments be ruptured; *a high state of inflammation would not supervene from a disruption of the ligaments of the wrist.* If there was a fracture and luxation of the joint in a person of Mrs. Ostram's age there would be a question whether she would recover; I think the probabilities against her complete recovery; this is a probable result, independent of the skill of the surgeon. There might be a fracture and no luxation. *I never heard of "Colles' Fracture of the Radius!"*

*Re-direct examination.*—If the bone gives way dislocation is not likely to occur; I perceived no luxation except of the ulna. Gross is best authority; Valpeau, by Mott, Errichson and Ferguson.

Oliver J. Ostram, the plaintiff, sworn, testified: Live in Buffalo; lived near Fort Erie in Canada, in 1862; am acquainted with defendant; in 1862 he was a physician and surgeon at Fort Erie; I called him in fall of 1862 to see my wife who had fallen and hurt her wrist; she was in her chamber and called me, and I went up stairs; she was sitting on the bed; she had been up stairs half an hour; when she went up she was not hurt, but when I went up I found her wrist hurt; I then went after Mrs. Forsyth, and then after defendant, and he came back with me; we returned within an hour; I went in room with defendant and he fixed her arm as soon as he could; have most forgotten what he did; can't remember; he put on splint on arm reaching from fingers to elbow and a bandage on it before he put splints on; I held her wrist and he pulled it and rolled it and then put on splints, and I can't tell what; the hand lay on the splint flat and straight and was so fastened and bandaged; Mrs. Forsyth and defendant were there; I believe he called it a dislocation; he told her to carry it in a sling; can't tell how or if anything was said as to how hand should be carried; he ordered sling for Mrs. Ostram and she had it; he came again on Sunday, (next day;) can't tell what he did; I had no help when he was there on Sunday; he made his last visit in February; before this accident my wife's health was good; she was doing her own work; she was about [illegible]0 [64] years old; it was the left arm that was injured; I moved from Canada in March, 1863; in February, 1863, had Dr. Learned and Dr. Elliott examine injury, and afterwards the two Drs. Dayton and Lothrop, in June, 1863; her hand was in bad condition, was weak, and she was unable to use it; has been weak ever since, and she has not been able to do work as before; she has worked with her right hand.

*Cross-examined.*—The Doctor put splints on both sides of arm; the one on top of arm short, and the one underneath reaching to hand; before he did anything he examined to see what the matter was; I held the wrist to help him draw it into its natural place; he then bandaged it; think he did not bandage hand before he put splints on; he bandaged wrist before he put splints on; I think he put splints on before bandage; think he said wrist was dislocated; he told her she could lay her arm on her lap; don't remember any pillow being used.

Sarah Forsyth sworn for plaintiff, testified: I live in Fort Erie and have lived there twenty-four years; was present when defendant was called and arm was dressed; he came, examined arm and proceeded to bandage it; he then put long splint on under side of arm supporting hand and then put a roll on to elbow; he did not give any opinion as to injury; hand was placed on a straight splint and bandaged; he ordered warm water to be used to wet arm, and to carry it in a sling, to be placed so the hand would pass through the sling; I remained until he went away; the wrist was much swollen on both sides at the time.

*Cross-examined.*—I was called about nine o'clock in evening and found Mrs. Ostram in bed up stairs; she seemed to be suffering; did nothing to relieve her until the defendant came there; was present all the time he was there; did not hear him say what injury was; when I got there I thought Mrs. Ostram was under the influence of liquor; my opinion is she was accustomed to be so; I then was her nearest neighbor; saw her frequently; I saw her when I thought her under influence of liquor; can't say that I saw her drink liquor; can't say plaintiff was under influence of liquor at the time; I don't recollect the defendant expressed an opinion as to how long this injury would last.

*Re-direct examination.*—I have seen Mrs. Ostram four or five times under the influence of liquor; I was at plaintiff's when defendant dressed wrist on first and second times.

Mary Ostram sworn for plaintiff, testified: I went home Sunday morning; when defendant came he undid arm and did it up; he said there would be no change in so short a time; asked him how long before she could use her arm; he said in four to six weeks; asked what it was; he said it was out of joint; did not loosen splints when he undid arm; he told me to loosen bandage when I bathed arm; she was to carry her arm as would make her most comfortable; don't remember as anything was said about a sling; he came again on Tuesday, loosened upper bandage and bathed with tepid water; arm and fingers were swollen; I stayed two weeks, and during that time he was called away; I took care of my mother while I was there; followed defendant's directions; when I left my sister, Mrs. Rice, and my cousin, Fanny Butler, took my place; saw no difference the two weeks I was there; it was two or three weeks before I saw it again; was no better then; saw her again almost three weeks after; 1 was present on another occasion when splints were taken off; this was on my third visit; the hand was swollen and stiff; was black and blue on inside back to the wrist; she had no power to move her fingers; saw her try to move them; defendant tried, but did not, as mother stopped him; he said it had not got along as well as he had expected in first place; said she would have use of her hand in six months; the wrist was swollen, but think you could see the joint; he directed to bathe it with liquor and liniment; after splints were removed mother carried her arm in a sling; she did before part of the time and defendant found no fault with it; when splint was removed he gave no reason why arm did not recover; I helped father and mother move and get settled, and then went down occasionally and baked and helped what I could; my sister died a year ago last November; mother first got use of her fingers three or four weeks ago; up to that time had no power over them; it was ten months or a year after injury before she could bring thumb and finger together, but had no strength; she can now clasp her hand, but hold no weight; can hold fork, but not with pressure; before injury she was a great hand to work; she does not sew now; can't say if she knits; have not seen her do it.

*Cross-examined.*—I went to father's in forenoon and defendant came soon after me; told me to loosen upper bandage; he did not tell me to loosen lower bandage nor to touch it; after his second or third visit he was called away; can't tell how long he was gone; he told me before he went to loosen upper bandage; did not want lower one disturbed; the arm stayed about as it was while he was away; I did not loosen lower bandage; mother set up during this time; she walked around; did not attempt any work; I stayed first time two weeks, then went away three weeks, then came and stayed a week, then went away and stayed two or three weeks; dressing not taken off but once while I was there as much as a week; Miss Bryan was there two or three weeks to do house-work; then Sarah Roth came and stayed awhile; she left day before father moved to Buffalo.

Lewis P. Dayton, sworn for plaintiff, testified: Have been a physician in Buffalo 20 years; have seen injury on Mrs. Ostram's arm; I saw it two years ago in spring; she lives in Black Rock; have examined it lately; there is a change in the hand since I first saw it; hand has become more limber. From my examination, I think there was a fracture of the lower end of radius—oblique fracture; from the outside oblique to inner side of bone; probably more or less injury to ligaments of joint. I think a surgeon could tell what the matter was; possibly he might be mistaken; the swelling might deceive him; a dislocation of the wrist joint would be an extraordinary occurrence; I should treat dislocation by extending ligaments and reducing; when reduced should bandage; might splint, or not; should keep arm in line; if fractured, should reduce and place on a curved splint, curving the hand to the ulna side; I should adapt dressing to circumstances, looking at well arm; with bandages to hold splints in place; should tell patient to place arm in sling, having weight rest not on hand but on arm.

Good surgeons dress arms on straight splints; I prefer dressing on crooked splints; my objection to straight splint is, there is more danger of shortening radius and throwing out the ulna; no other objection; any displacement of bones will weaken bones, though we have cases where broken bones are as strong as ever; I do not think the bones in Mrs. Ostram's arm in the right place; there is, at any rate, a prominence of the ulna; the hand is not perfect; there is a stiffness of fingers produced by injury; there are cases of this sort that have been adjusted where there is no such results; have treated cases of fracture of radius—four or five cases; I don't know that any of them show this condition; in my cases they recovered power of hand in from a few months to years; to keep power in hands I have recommended friction and motion; I have seen cases where motion was very little on removal of splints; never saw perfect anchylosis, but very near; I had one case—patient 71 years old—when I took splints off she could move fingers a little; slight prominence of ulna; she got use of hand after two years; improvement was gradual; don't remember ever seeing case like this one; this arm has not been restored to its perfect condition; I think defendant did not accomplish what he intended; did not obtain expected results; I would not dress hand on straight splint; should use curved splint—to succeed best I could; don't think straight splint as good as curved; if hand was curved on splint it might answer same ends; I have always used curved splint, and *have no experience with the straight splint.*

*Cross-examined*—I have treated four or five cases of fracture in all; have used curved splint; I have not obtained as good results as expected; two occurred with old persons; did not recover as soon as I expected; in both cases anchylosis was great in wrist and hand, and of long continuance; in one case two years after there was a good deal of anchylosis of the whole hand; in the other case it did not continue so long—say 18 months to two years, and I do not think it perfect yet; the lady is about 52 years old; it was Mrs. Gordon, at Black Rock; I do not think this attributable to mal-practice; she uses the hand about her work; this injury occurred thirteen years ago; she also had a fracture of the shaft of the radial bone; have seen no cases in charge of other physicians; in a fracture of this kind there would be displacement in some degree; it would produce inflammation in a case of this kind; *I do not consider this wrist as evincing bad practice on the part of the attending physician.*

*Re-direct examination*—When there is displacement it takes longer to recover.

Nancy Purrington sworn for plaintiff: Have known Mrs. Ostram eight or nine years; visited her during that time; saw her first after injury after she had moved from Canada; she was healthy and a hard-working woman; have visited her at Black Rock; her daughter, Mrs. Riel, got tea for us; she had no use of her left hand; have never seen her when she had drank too much.

*Cross-examined*—I am related to her by marriage on husband's side; my acquaintance was more with children than old lady.

Mary Ostram, recalled by plaintiff: Never saw mother under influence of liquor.

Plaintiff here rested her case.

Defendant's counsel then moved the court for a non-suit, on the ground that the plaintiff had failed to make out a cause of action; 2. That there was no evidence of mal or other improper practice on part of defendant shown.

The court denied the motion, and defendant's counsel excepted.

Sandford Eastman, sworn on behalf of defendant, testified: I reside in Buffalo; am a physician and surgeon in practice, and have been for fifteen years in May next; I practice surgery more than average of profession; have treated fracture of radius and ulna; have a good many cases; it is usual to treat with splints on front or back of arm and hand; some advise splint on one side, some on the other, and some on both sides; some advise stʌaight splints, some curved splints; I think a straight splint good treatment; it is recommended by best authority in this country; good cures can be expected by use of straight splint; Dr. GROSS recommends straight splints; he is among the best authorities; Dr. HAMILTON recommends straight splints; Dr. SMITH, of Dublin, also, and Dr. MOTT does the same; I should consider it good practice to dress by bandaging hand and applying splints and putting on permanent bandage. In all situations the muscles are strained by fracture; there would be more tension if the fall was on the back than on the palm of the hand; in a person of this age (64) there would be uncertainty in the re-contracting of ligaments; the ligaments lose their contractile power; good practice to put arm in sling; it would not be improper to direct that the arm rest on pillow or table. In fractures of radius there is almost always displacement of ulna; it is usual to have stretching of the tendons in these cases, and consequent stiffness after such injuries; there is always stiffness of parts; it is depending on the injury and its consequences; it acts on the entire hand; much may be due to want of motion; this want of motion is necessary to a cure; it remains a longer or shorter time; have known it in one case two years after, and last time I saw her the hand was stiff; our books teach us that this often remains for years, and even for life, in cases well treated; it is alwasy longer with old than young people; the prominence of the ulna often remains in spite of best treatment and most skillful attention. The straight splint is the splint I use.

*Cross-examined.*—In half the cases I have had, I have not succeeded in reducing ulna to a line; I have succeeded in giving motion; I never saw dislocation of wrist joint; I have had difficulty in the early part of my practice, in determining between a fracture or dislocation; the muscles on the back of hand have nothing to do with motion of hand; they are on the palmer side; the ulna bone has nothing to do with motions of hand, it depends on muscular action, the projection of the ulna has nothing to do with it; I use a straight splint—a combination of the Bond and Swinburn splint; I cannot say, if hand was swollen and black and blue, it was caused by bad practice.

Julius F. Miner, sworn for defendant, testified: Reside in Buffalo; am a surgeon; have been in practice 20 years; have made surgery a specialty; have practiced it almost exclusively as a business for last six years, in amount unsurpassed by any other in the State outside of New York city; have frequently been called to treat fractures of radius; during the past winter I have treated seven cases in one week; I have seen and examined Mrs. Ostram's arm; I think no surgeon can determine the exact injury by present examination; cannot tell exact location or amount of displacement, if comminuted or not; my impressions and best knowledge are, there was a fracture of lower end of radius; that it extended into articulation or not, can't say, or if commiunted can't say; I think the lower extremity of radius has been a little shortened, and perhaps approximated to ulna, and as the radius constitutes almost the entire base on which the carpus rests, it changes the direction of the hand—draws hand to radial side and renders ulna prominent. If body fell upon hand it would be more likely to displace ligaments than if no weight had fallen on it; it would be proper, in dressing such an injury, to bandage hand, and put on splints with permanent bandage; in my judgment there is no difference whether you use a straight or pistol splint; it would be a proper direction to place arm in a sling; that would place hand in a condition between pronation and supination; there is almost always a deformity in this fracture, which has led the profession to give great attention to it; there is more liability to stiffness and disability with old people than young; after removal of splints there is almost always a great amount of stiffness; people of Mrs. Ostram's age recover much slower than young people; that is owing to causes wholly beyond control of surgeons; I have case where I am not aware they have recovered any more than the one in question; this case I dressed with Dr. Barnes and there is as much disability as with this lady; this is a case where there is but little if any deformity; I have other cases where time is shorter and the inability considerable; there are cases of this sort where recovery is after some years; I see nothing in wrist that is evidence of improper treatment on part of surgeon; I should infer it was owing to causes beyond the control of attending surgeon.

*Cross-examined.*—If I had seen injury within an hour, think I could tell what was the matter; there might have been dislocation or not; I think dislocation of wrist joint very uncommon; when there is fracture of the radius there is greater liability to rupture ligaments that attach the ulna to carpus; in Mrs. Ostram's arm the ulna is in its natural position; the radius is thrown down upon ulna. I believe a fracture may be as well dressed on straight as crooked splint; I don't remember that I have a case where hand slides off as much as Mrs. Ostram's; if it recovers in motion and use, it will become more natural in form; I think there is uncertainty whether she will recover her hand; have had case where fingers could not be moved at end of six weeks; had case where the hand could not be closed in four or five months; this was a lady about 45 years old.

*Re-direct.*—Sprains or injuries often allow hand to slide and produce results like this; this sometimes happens without injury.

John Cronyn sworn for defendant, testified: Reside in Buffalo; have been a practicing physician and surgeon sixteen years; have seen and examined Mrs. Ostram's arm; its present condition evidences a fracture at lower extremity of radius; there are three kinds of fracture; the one in this case seems to be an impacted fracture, oblique; this is most common; I place bandage over splints, not on arm, though some do; it is not improper to do up arms in straight splint; in the many cases I have had I never but once used any other than staight splint; in such cases have known good results; once I put arm in pistol splint; I got no better results; it is proper to dress with either; the stiff state of fingers continues a long time, from months to years; in one case like this Dr. Hamilton treated it with similar results to one in suit; I had care of same case afterwards.

*Cross-examined.*—If I had been called within an hour could have told what injury was.

Sarah Forsyth re-called by defendant: While plaintiff lived on farm near me, he had a hired girl; this was while ordinary family was there, one girl only; they had hired girl spring, summer and part of fall of 1862; no hired girl previous; Mrs. Rice and Miss

Ostram were there; I was in frequently to plaintiff's after accident; in February following Mrs. Ostram began working round the house; she had no hired girl at that time; they moved about March 1st; after they moved in July, 1863, I visited Mrs. Ostram in Black Rock; she seemed to be well; heard no complaint; she was engaged about ordinary duties of house-keeping; she had just been baking and taking bread from the oven; plaintiff had no hired girl that I saw; was there from one-half to an hour; plaintiff was there when I got there; before they moved from Canada I went in and found Mrs. Ostram washing dishes; she used her left hand on that occasion; she did other light work about the house, such as she could do; they lived on farm three or four weeks, after injury, without a servant.

*Cross-examined.*—Plaintiff carried on farm at Fort Erie; Colburn was with him; he had help occasionally.

Peter Tertius Kempson sworn, testified: I am defendant in this action; reside at Fort Erie; am physician and surgeon; was called upon by plaintiff to attend his wife for injuries to arm; I went: when I got there I found Mrs. Ostram in bed, examined arm, found it displaced, and a fracture of lower end of radius; as near as I recollect I used extension and counter-extension, and having brought parts in position I proceeded to swathe wrist and laid on cloths wet in warm water, while preparing splints and bandages; after all was ready placed arm in position—placed it on a padded splint, and another on the top; I then bandaged the arm up nearly to elbow; I directed patient to use a sling and to keep arm in it; I allowed her to rest arm on pillow that night; think I placed pillow for her and left it; I gave directions to lay rags wet with warm water over arm; I had no doubt about injury at that time; I next saw arm on following morning; I inspected dressing and arm; found everything was regular and straight, as far as I could then discover; I saw it next morning; all right then; I went as often as I thought necessary—not as often as I should but they seemed afraid of expense; I was over-confident I reduced it as well as it could be reduced; before this I had experience, some in Canada, and prior in England, from whence I came to Canada; my experience in England had been considerable in these fractures; I dressed this arm on a straight splint; I did not tell them—the plaintiff or his family—that this was a sprain or dislocation of the wrist; I gave them as hopeful an account as I could; I did not assure them a perfect arm; I state this positiveiy; I told them she would be able to use arm in six or eight weeks; I think I removed the top splint in five or six weeks; can't tell who was there; think Mrs. Forsyth was; the arm was then going on quite as well as I expected; the arm was then in its natural position, except the ulna, which projected; she had then no use of arm; I did not allow it; the arm was stiffened, but she had some use of her fingers; took off bottom splint soon after; the permanent splints were removed at end of six weeks; there was then a perfect state of arm except a little deformity, but had this stiffness; can't tell if I left it in bandage; recommended lotion and embrocation to overcome stiffness; the last time I saw him was February 17, 1863; the condition of the arm was then much as when I left it before; the ulna did not project as it does now, and there was no deformity.

*Cross-examined.*—I came to Fort Erie April 7, 1854; did not put out sign until four or five years after; I practiced when called on; did not practice generally; I farmed some; I remember two cases of fracture of radius besides this; these cases were both in young persons, not like Mrs. Ostram; one was a child of Mr. Rose, the other a child of Mr. Kirtz; I lived at Brierly Hill in England; Mrs. Forsyth assisted in dressing the arm.

The defendant rested his case.

The case was then submitted to the jury by the counsel for the respective parties, and thereupon the Court charged the jury substantially as follows:

*Gentlemen of the Jury:*—I shall consume but a short time in submitting this case to you. I shall generalize it, and leave it with you to pass upon. In November or December, 1863, the wife of plaintiff fell and fractured the radial bone near the articulation of the hand. There is no dispute as to the injury, nor in respect to the present condition of the wrist. The evidence is that the radius is some shorter than the ulna. Injury and treatment have resulted in deformity with stifiness of fingers. Dr. Miner says, it is hardly probable that it will entirely recover. The theory of the plaintiff is, and here is the point of departure between the counsel, that the defendant said at the time of injury, that the arm was out of joint, and the plaintiff claims the defendant did not suspect fracture, and therefore did not reduce it. Defendant says he did treat it as a fracture, and that he reduced and put it in place. There is no dispute about the manner of dressing arms, but plaintiff claims defendant's treatment was not proper, and calls Drs. Hill and Dayton, who say they use crooked splints which produce best results; that the reason is, by curved splint you get strain on radial muscles—you get tension on muscles that hold hand in place.

The defense says the curved splint is good, but that it is not improper to use straight splints, and that as good results are produced by them as by crooked splints. The evidence of defendant's witnesses is, that present condition of arm is result of injury, and not of treatment. If you rely upon the testimony of defendant's witnesses your verdict should be for the defendant. If you rely on the evidence of the plaintiff's witnesses then your verdict should be for plaintiff, and you will then consider what damages he should recover. All you can give is for plaintiff's pecuniary loss up to present time on account of the loss of service of his wife in consequence of improper treatment by the defendant, if you find his treatment was improper, and you will not take into account the injury either to feelings or person.

The jury rendered a verdict for plaintiff for $50. A stay of proceedings was granted by the Court, and the case will be carried up for review.

Perry G. Parker and Charles J. Thomas for plaintiff.

W. W. Mann and George W. Cothran for defendant.

